# Antimicrobial Resistance (AMR) of Bacteria Isolated from Dogs with Canine Parvovirus (CPV) Infection: The Need for a Rational Use of Antibiotics in Companion Animal Health

**DOI:** 10.3390/antibiotics11020142

**Published:** 2022-01-23

**Authors:** Giorgia Schirò, Delia Gambino, Francesco Mira, Maria Vitale, Annalisa Guercio, Giuseppa Purpari, Francesco Antoci, Francesca Licitra, Gabriele Chiaramonte, Maria La Giglia, Vincenzo Randazzo, Domenico Vicari

**Affiliations:** Istituto Zooprofilattico Sperimentale della Sicilia “A. Mirri”, Via Gino Marinuzzi n. 3, 90129 Palermo, Italy; giorgia.schiro91@gmail.com (G.S.); deliagamb@gmail.com (D.G.); maria.vitale@izssicilia.it (M.V.); annalisa.guercio@izssicilia.it (A.G.); giuseppa.purpari@izssicilia.it (G.P.); francesco.antoci@izssicilia.it (F.A.); francescalicitra15@gmail.com (F.L.); gabrielechiaramonte90@gmail.com (G.C.); maria.lagiglia@izssicilia.it (M.L.G.); vincenzorandazzo78@gmail.com (V.R.); domenico.vicari@izssicilia.it (D.V.)

**Keywords:** dog, canine parvovirus, *Carnivore protoparvovirus 1*, antimicrobial resistance, multidrug resistance, One Health, *Enterobacteriaceae*

## Abstract

Canine parvovirus type 2 (CPV-2) represents a major viral threat to dogs. Considering the potential effects of pets on antimicrobial resistance, information on the CPV and associated bacterial co-infections is limited. The aim of this study was to analyze the antimicrobial susceptibility and multidrug-resistance profiles of bacterial species from tissue samples of dogs with canine parvovirus infection. A set of PCR assays and sequence analyses was used for the detection and the molecular characterization of the CPV strains and other enteric viruses. Bacterial isolation, the determination of antimicrobial susceptibility via the disk diffusion method, and the determination of the minimum inhibitory concentration were performed. The detection of β-lactamase genes and toxin genes for specific bacteria was also carried out. CPV infection was confirmed in 23 dogs. Forty-three bacterial strains were isolated and all showed phenotypic resistance. Seventeen multidrug-resistant bacteria and bacteria with high resistance to third- and fourth-generation cephalosporins and metronidazole were detected. Almost 50% of the isolated *Enterobacteriaceae* were positive for at least one β-lactamase gene, with the majority carrying more genes as well. The evidence for multi-resistant bacteria with the potential for intra- or cross-species transmission should be further considered in a One Health approach.

## 1. Introduction

Canine parvovirus type 2 (CPV-2) emerged as a dog pathogen in early 1978 and rapidly spread worldwide, causing a pandemic event [1]. Despite the widespread use of effective vaccines, after forty years, CPV remains a significant pathogen, still representing a major threat to young dogs [2,3,4].

Canine parvovirus (CPV) is a small (about 25 nm in diameter), non-enveloped DNA virus, recently included with other related parvoviruses in the unique species *Carnivore protoparvovirus 1*, within the *Protoparvovirus* genus (family *Parvoviridae*, subfamily *Parvovirinae*) [5,6,7]. Soon after its emergence, two antigenic variants (CPV-2a and CPV-2b) were identified, replacing the original CPV-2 type [8,9]. In 2000, a third antigenic variant (CPV-2c) rapidly spread, and all three variants are currently distributed worldwide with different prevalence rates [5,10].

Parvoviral infection is characterized by depression, anorexia, vomiting, and severe enteritis; mucoid or bloody diarrhea, dehydration, leukopenia, fever, and shock are also detected [5,11]. No specific therapy exists for CPV, and therefore, treatment is primarily supportive and symptomatic, mainly based on rehydration and antimicrobial and antiemetic therapies [11,12,13].

Alterations of the intestinal mucosa have been associated with CPV infection [14], leading to the disruption of gut barrier function and microbiota dysbiosis [15,16,17]. These changes can result in bacterial and endotoxin translocation, with the consequent development of systemic inflammatory responses and multiple organ dysfunction syndromes [11]. A broad spectrum of antibiotics is used in the therapy of CPV infection, although the use of antibiotics may cause an increase in the release of endotoxins and/or the exacerbation of the systemic inflammatory response [14,18]. Despite the close relationship between CPV infection and the bacterial population, few studies on these co-infections are currently available [19,20,21,22].

Moreover, the diffuse and sometimes uncontrolled use of antibiotics in veterinary medicine has increased concerns related to the high diffusion of antimicrobial resistance (AMR), a real threat to public health in all countries. For the efficacious control of AMR spread worldwide, a One Health perspective has been suggested by the international authorities for public health [23]. Particularly, attention is focused on those critically important antimicrobials (CIAs) for human medicine [24]. Indeed, there is considerable evidence that their use in animals can also contribute to antimicrobial resistance among some common enteric human pathogens [25,26]. Most of this renewed attention is focused on food-producing animals, as well as their potential role in environmental contamination with AMR strains; however, a small—but increasing—amount of the current research is also looking at the potential role of pets.

The aim of this study was the evaluation of the antimicrobial susceptibility and multidrug resistance (MDR) profiles of bacterial species from the tissue samples of 23 dogs with canine parvovirus infection. Co-infection with other viruses was also analyzed.

## 2. Results

### 2.1. Clinical Cases

All 23 sampled dog carcasses, with a modal age value of 2 months (ranging from 40 days to 2 years), were submitted for suspected infectious gastrointestinal disease. Most of them (n = 16) were young dogs (<12 months) and mixed breed stray (n = 15) dogs (Supplementary Materials Table S1). Anamnesis, the clinical history, and the vaccination statuses of most of the analyzed dogs were unavailable.

At necropsy, the common anatomopathological lesions characteristic of CPV infection were observed: hyperemia of the gastric mucosa and catarrhal-hemorrhagic fluids in the stomach, hyperemia, and hemorrhage of the serous membrane of the small intestine, and congestion and enlargement of mesenteric lymph nodes. In some dogs, further lesions (brain edema and hemorrhage, paleness of and focal fibrous lesions on the myocardium, ecchymoses, petechiae and necrosis on the lungs, subcutaneous petechiae, and icterus and hepatic lipidosis) in different organs were observed (Supplementary Materials Figure S1).

### 2.2. Viral Detection and Molecular Characterization of CPV

All the tissue samples tested positive for CPV by conventional PCR assay. Positive samples were obtained both from commonly and from less tested tissues, such as the brain and cerebellum, bone marrow [27], and spinal cord. The tissue samples tested negative for canine distemper virus (CDV), canine adenovirus (CAdV) type 2, and canine rotavirus (CRoV), with the exception of five intestines, which tested positive for canine coronavirus (CCoV), and the tissues of dog id. 19, which tested positive for CAdV type 1 (Supplementary Materials Table S1).

Based on the analysis of the VP2 amino acid residues, 9, 3, and 11 CPV strains were typed as CPV2a, CPV-2b, and CPV-2c variants, respectively.

The phylogenetic analysis (Supplementary Materials Figure S2) evidenced the relationship with CPV-2a/2b/2c strains previously reported in Italy [28,29], as well as with CPV-2c strains more recently circulating in Italy and in Asia [30,31]. The viral variants are listed in Supplementary Materials Table S1.

### 2.3. Bacterial Detection

The bacteriological examination was carried out on 161 tissue samples. One or more bacterial species were isolated from all the dogs, for a total of 43 strains, mainly from the intestine but also frequently from the brain, liver, spleen, heart, kidney, and, less frequently, lung and lymph nodes (Table 1, Supplementary Materials Table S1). The most isolated bacterial species (n = 31) belong to the Gram-negative group (72%), with the highest prevalence at the species level represented by *Escherichia coli* (19/43, 44%). *Klebsiella pneumoniae* (4/43, 9.3%), *Enterobacter* spp. (4/43, 9.3%), *Escherichia fergusonii* (1/43, 2.3%), *Salmonella enterica* subsp. *Enterica* serovar Schleissheim (1/43, 2.3%), *Proteus mirabilis* (1/43, 2.3%), and *Pseudomonas aeruginosa* (1/43, 2.3%) were also detected. Among the Gram-positive bacteria (n = 12), equal amounts of strains belonging to the genuses *Enterococcus* (3/43, 6.9%), *Staphylococcus* (3/43, 6.9%), *Streptococcus* (3/43, 6.9%), and *Clostridium* (3/43, 6.9%) were isolated.

A unique bacterial species was isolated in 43% (10/23) of the dogs, whereas in 22% (5/23) and 26% (6/23), two and three bacterial species were simultaneously isolated, respectively. The coexistence of four different bacterial species was detected only in one dog (id. 20). Although they are normally present in the intestinal microbiota, *E. coli* strains were isolated from the intestines of only 7/23 dogs. In 12/23, *E. coli* was also isolated from other organs. The isolated bacterial species are listed in Table 1 and Supplementary Materials Table S1.

### 2.4. Antimicrobial Susceptibility According to the Disk Diffusion Method

The results obtained with the Kirby-Bauer method showed the presence of resistance in all 43 isolated strains. All the Gram-negative strains (31/43, 72%) were resistant to cefquinome (fourth gen. cephalosporin), methicillin, and metronidazole. Some strains were resistant to antibiotics considered the last line of defense against resistant infections in human health: *Escherichia fergusonii* was resistant to colistin sulphate, one *E. coli* to imipenem, and three *E. coli* were resistant to chloramphenicol. Two more *E. coli* with two *Klebsiella pneumoniae* strains and one *Enterobacter cloacae* strain were resistant to ceftriaxone. Two *E. coli* isolated from dogs 19 and 22 were simultaneously resistant to chloramphenicol/ceftriaxone and chloramphenicol/imipenem, respectively. Additionally, another five bacteria (four *E. coli* and a *Pseudomonas aeruginosa*) showed intermediate sensitivity to imipenem, and one more *E. coli* isolate to chloramphenicol. In addition, many of the strains of *Enterobacteriaceae* were resistant to cefadroxil and cephalexin (first gen. cephalosporin). All the *Klebsiella pneumoniae* (4/31), one of two *Enterobacter gergoviae* (2/31), and *Salmonella enterica* (1/31) were found to be resistant to cefadroxil and cephalexin, and one *Enterobacter gergoviae* (2/31) was resistant to cefadroxil and sensitive to cefalexin, while of the 19 *E. coli* isolated, 15 were resistant to cephalexin and 14 to cefadroxil.

On the other hand, most of the Gram-negative strains were sensitive to marbofloxacin (24/31, 77.4%), enrofloxacin, and sulfamethoxazole/trimethoprim (19/31, 61.2%). Despite the reported intrinsic resistance [32], one *Enterobacter cloacae* and the *Pseudomonas aeruginosa* strains tested sensitive to penicillin, chloramphenicol, and doxycycline. The antibiotic sensitivity results for the Gram-negative strains are shown in Table 2.

Most of the Gram-positive strains were resistant to metronidazole (10/12, 83.3%), cefquinome (fourth gen. cephalosporin) (9/12, 75%), and cefuroxime (second gen. cephalosporin) (6/12, 50%) and sensitive to vancomycin (12/12, 100%), amoxicillin/clavulanic acid, and imipenem (10/12, 83.3%), marbofloxacin (8/12, 66.6%), cephalexin, cefadroxil, and ceftriaxone (first and third gen. cephalosporin, respectively) (7/12, 58.3%). The antibiotic sensitivity results for the Gram-positive strains are shown in Table 3.

### 2.5. Antimicrobial Susceptibility According to the Minimum Inhibitory Concentration (MIC)

To quantitatively assess the bacterial sensitivity to some of the antibiotics previously tested with the Kirby–Bauer test, five sets of antibiotics were used to determine the MICs; additional molecules were selected by the manufacturer according to the bacterial species. The MICs are reported in Supplementary Materials Tables S2 and S3.

The set used for the 28 Gram-negative bacteria confirmed the higher incidence of resistance to cephalexin (77.7% of the 27 strains tested) and ampicillin (47.3% of the 19 strains tested): 15/28 *E. coli*, 2/28 *Enterobacter cloacae*, 2/28 *Enterobacter gergoviae,* and 2 *Klebsiella pneumoniae* were resistant to cephalexin (first gen. cephalosporin) and 9/28 *E. coli* to ampicillin. Some antibiotics were tested only by the MIC assay, and the following results were derived: 8/28 *E. coli*, 4/28 *Klebsiella pneumoniae,* and 1/28 *Enterobacter cloacae* were found to be resistant to piperacillin (46.4%); 6/28 *E. coli*, 4/28 *Klebsiella pneumoniae*, 2/28 *Enterobacter cloacae,* and 1/28 *Proteus mirabilis* to tetracycline (48.1% of the 27 strains tested). All of them were sensitive to amikacin, and many, to marbofloxacin (24/28, 85.7%), tobramycin (22/28, 78.5%), enrofloxacin, and gentamicin (21/28, 75%) as well. The two isolates, *E. fergusonii* and *S. enterica,* showed sensitivity to all the tested antibiotics.

Among the Gram-positive strains, the MICs determined for 1/8 *Streptococcus pseudoporcinus* showed sensitivity to all the tested antibiotics, whereas 1/8 *Streptococcus canis* was resistant to tetracycline only. However, resistance was found in the strains of *Enterococcus faecium* and *Staphylococcus xylosus*: both *Enterococcus faecium* strains tested were resistant to enrofloxacin, marbofloxacin, and doxycycline, and one of them, also to erythromycin, while the *Staphylococcus xylosus* strain tested was resistant to clindamycin, enrofloxacin, marbofloxacin, doxycycline, and minocycline. Moreover, all the Gram-positive strains have been found to be sensitive to chloramphenicol and florphenicol.

### 2.6. Molecular Analysis of β-Lactamase Genes

All the *Enterobacteriaceae* (30 isolates in Table 4) from the dogs were further analyzed for the presence of β-lactamase genes. The results show that almost 50% (15/32) of the isolates were positive, according to PCR, for at least one β-lactamase gene, with the majority also carrying more genes simultaneously. Of the 15 β-lactamase-positive strains, 11 isolates carried the *bla*_TEM_ gene and nine the *bla*_CTXM-II_ gene.

Some isolates of *E. coli* tested positive for at least one β-lactamase gene (8/19). All but one carried the *bla*_TEM_ gene, mostly associated with other genes. Only one carried just *bla*_OXA_ and *bla*_CTX-M-II_, and one, only *bla*_TEM_ (Table 4). *Klebsiella pneumonia* was also isolated from four mixed breed dogs (three strays and one owned). Of these isolates, two carried four β-lactamase genes simultaneously (*bla*_SHV_, *bla*_OXA_, *bla*_TEM,_ and *bla*_CTXM-II_), one carried three genes (*bla*_SHV_, *bla*_TEM,_ and *bla*_CTXM-II_), and one carried only the *bla*_SHV_ gene, normally present in all *K. pneumonia* strains.

The unique isolate of *Salmonella enterica* tested negative for the presence of the β-lactamase gene, confirming the sensitivity of this strain in the MIC assay, in contrast to its ampicillin resistance as determined by the KB method. The unique isolate *E. fergusonii* was sensitive to all the antibiotics, although the *bla*_CTX-M-II_ gene was present according to PCR. Of the *Enterobacter* spp., represented by two *gergoviae* and two *cloacae*, one *E. gergoviae* was positive for *bla*_SHV_ only, and one *E. cloacae*, for the *bla*_TEM_ and *bla*_DHA_ genes (Table 4).

### 2.7. Molecular Analysis for Virulence Factors in Enterobacteriaceae, Staphylococcus spp. and Clostridium perfringens (A to E)

All the *E. coli* (19 isolates) were analyzed for the presence of genes that code for serogroup-specific O-antigens and four major virulence factors (intimin, enterohemorrhagic hemolysin, and Shiga toxins [Stx] 1 and 2), to detect O157, O26, O45, O103, O111, O121, and O145. The four virulence factors were also studied for all the other strains of *Enterobacteriaceae* (11 isolates). All the strains tested negative for the four virulent genes, except for one *E. coli* strain from dog id. 9, which carried the eae and the serogroup O111 genes (Table 4).

The *Staphylococcus* spp. strains showed negativity for the mecA and enterotoxin genes, except for *S. xilosus* (dog id. 4), which carried the enterotoxin D gene. One *Clostridium perfringens* strain (dog id. 3) showed the presence of the cpa gene, encoding the alpha-toxin.

### 2.8. Multidrug-Resistance Evaluation

To better assess the presence of multidrug-resistant strains among the 36 isolates tested using both methods (Kirby-Bauer and MIC), the results obtained with the two methods for the different antibiotic classes were compared. However, since the cards used to determine the MIC with VITEK^®^ contain predetermined antibiotics, it was not possible to test the same molecules with both methods. For this reason, for the beta-lactam and tetracycline classes only, the comparison was based on different molecules of the same class, and for some other molecules (i.e., metronidazole), the comparison was not possible.

Among the Gram-negative strains, for the 27 *Enterobacteriaceae*, both methods confirmed sensitivity to the chloramphenicol class. Few variations were found between the two methods for penicillin and sulfonamides, while clearer differences emerged for cephalosporins, beta-lactams, aminoglycosides, fluoroquinolones, and tetracyclines (Table 5). The *Pseudomonas aeruginosa* strain was proven sensitive to all the antibiotic classes.

For the Gram-positives, no differences were found between the two methods for two of the eight *Streptococcus* spp., and the resistance of one strain to the tetracycline class was confirmed. For three of the eight *Enterococcus* spp., sensitivity to the chloramphenicol class was confirmed, but differences were evidenced for the fluoroquinolones and tetracyclines. For three of the eight *Staphylococcus* spp., both methods confirmed sensitivity to the aminoglycosides, chloramphenicol, and sulfonamides classes, and the resistance of only one strain to the lincosamides and fluoroquinolones. With MIC evaluation, the resistance of one strain of *Staphylococcus* spp. to macrolides was not confirmed, whereas resistance to the tetracycline class in one strain was observed.

Due to the variations among the methods, the results of the MIC method were considered to limit the overestimation of antimicrobial resistance. Bacterial strains showing resistance toward three or more antimicrobial classes were considered multidrug-resistant (MDR).

As a result of this comparison, 17 (47.2%) of the strains tested with both methods (15/27 *Enterobacteriaceae*, 1/2 *Enterococcus faecium*, and 1/1 *Staphylococcus xylosus*) were considered multidrug-resistant (Supplementary Material Tables S4 and S5). In particular, five strains of different bacterial species (1/16 *E. coli*, 1/4 *Klebsiella pneumoniae*, 1/2 *Enterobacter cloacae*, 1/2 *Enterococcus faecium,* and 1/1 *Staphylococcus xylosus*) showed resistance to three antibiotic classes; 1/16 strain of *E. coli* showed resistance to four antibiotics classes; seven strains (4/16 *E. coli*, 2/4 *Klebsiella pneumoniae,* and 1/2 *Enterobacter cloacae*), to five antibiotics classes; 2/16 strains of *E. coli*, to six antibiotics classes; and two strains (1/4 *Klebsiella pneumoniae* and 1/16 *E. coli*) showed resistance to seven and eight classes, respectively.

The multidrug-resistant strains were isolated from 13 dogs, and in one case, the presence of MDR was shown in all the strains isolated from the same dog. Indeed, the two strains, *Klebsiella pneumoniae* and *E. coli*, isolated from the stray dog id. 22 were resistant to seven (penicillin, cephalosporins, beta lactams, sulfonamides, fluoroquinolones, aminoglycosides, and tetracyclines) and five (penicillin, fluoroquinolones, aminoglycosides, chloramphenicol, and tetracyclines) classes, respectively. The same two bacterial species from dog id. 22 tested positive for the presence of the same four β-lactamase genes (Table 4). *E. coli* from dog id. 4 was resistant to five classes (penicillin, cephalosporins, beta lactams, sulfonamides, and tetracyclines), while *Staphylococcus xylosus* was resistant to three classes (tetracyclines, fluoroquinolones, and lincosamides); in the molecular analysis for the β-lactamase gene, the *E. coli* strain from dog 4 showed the presence of *bla*_TEM_ and *bla*_CTX-M-II_ genes (Table 4). In dog id. 10, the *E. coli* strain was resistant to eight antibiotic classes (penicillin, cephalosporins, beta lactams, sulfonamides, fluoroquinolones, aminoglycosides, tetracyclines, and chloramphenicol), although it was negative for the presence of β-lactamase genes when assessed by PCR, in contrast to the *Klebsiella pneumoniae* isolate, which was shown to be positive for the *bla*_SHV_ gene and was resistant to three classes (penicillin, beta lactams, and tetracyclines). Multidrug-resistant strains of *E. coli* (the species isolated with the higher rate) were isolated from the intestines of three dogs and from the intestines and other organs of another five dogs.

## 3. Discussion

Despite the fact that vaccination has considerably reduced the occurrence, canine parvovirus infection remains a global threat to domestic and wild carnivores. Until now, studies have been focused on CPV infection and global spread, with limited studies on co-infections with bacteria or other viruses [17,20,33,34]. In this study, samples collected from dog carcasses with CPV infection were analyzed to evaluate the impact of the bacterial species, their susceptibility to antibiotics, and their multidrug resistance, along with other viral co-infections. In total, 18 dogs were strays, three were owned, one was housed in a city shelter, and one was just imported from an Eastern European country. The lack of any specific therapeutic treatment or previous vaccination for stray dogs and the potential stressful conditions for the others could have contributed to the fatal infection outcome.

The occurrence of CPV infection has been mainly reported in young dogs, probably related to the lack of specific and protective immunization or stressful conditions [5,35]. The vaccines currently used for CPV are safe and effective and cross-protect against all three variants [3,36,37]. Their rational use [38], together with appropriate sanitation procedures [39], still represents the most effective protective approach to preventing viral infection [40]. Moreover, vaccines could also reduce the unnecessary use of antimicrobials, as recently suggested [41,42].

The most commonly described pathological findings for CPV infection [5] were observed in all the carcasses during the anatomopathological examinations. However, other lesions not specifically associated with CPV were also observed. These anatomopathological observations might suggest a mixed pathological pattern, potentially due to bacterial co-infections, the effect of the bacterial toxins, or the systemic inflammatory response (SIRS) [14,43,44]. The data in this study suggest the need for more specific assays, particularly in the extra-intestinal organs from which bacteria were isolated, to assess their role and/or the roles of their toxins in determining pathological lesions. Additional studies are also necessary to evaluate CPV’s action in extra-intestinal sites, such as the central nervous system (CNS) or bone marrow.

Although in a few dogs (id. 6, 7, 11, 21, 22, 19) co-infections with other viruses (CCoV and CAdV-1) were assessed, the results confirm that CPV infection remained the main cause of viral enteritis and acute hemorrhagic diarrhea syndrome (AHDS) in young dogs [33,34]. Therefore, a diagnostic panel for the main pathogenic bacteria and multiple viral agents should be considered in dogs with suspected infectious gastrointestinal disease [21,34].

From all the samples, forty-three bacterial strains were isolated, with a prevalence of Gram-negative groups and, particularly, *E. coli* species, isolated mainly from the organs of the gastrointestinal tract (liver, spleen, and intestine) and, less frequently, from extraintestinal organs. Furthermore, few species belonging to the Gram-positive group were isolated. Bacterial isolation from intestinal tissues was performed in five dogs only, while, in the others, bacteria were isolated mainly from the brain but also other tissues, suggesting a systemic or multi-organ infection. The evidence in this study of toxin-producing bacteria, such as *Clostridium perfringens*, and of other pathogenic bacterial strains in the brain suggests their role in the development of the neurological clinical signs, such as depression, commonly observed in live dogs with CPV infection [5,14,45]. However, further studies on the potential association of CPV with neurological lesions, as suggested in other reports [34,46,47,48,49], are necessary.

According to the results, the risk some bacteria pose of fatal outcomes in dogs with CPV infection appears to be partially limited. Indeed, most of the isolated bacteria (*E. coli* strains) represented normal intestinal flora and the fact that their presence was restricted to the enteric tract confirms their limited role in the pathogenesis of the dogs. Therefore, antibiotic therapy would most likely not have been necessary in these cases. Conversely, in eleven dogs, *E. coli* was isolated from extra-enteric organs, and in two dogs, bacteria harboring toxin genes (*Clostridium perfringens* and *Staphylococcus xylosus*) were also evidenced. In almost half of the analyzed canine carcasses, two or three different bacterial species were isolated, suggesting their potential role in developing the clinical signs and contributing to the *exitus,* or the observed pathological findings, as previously suggested [21,50]. In these cases, antimicrobial therapy in vivo might have been suggested and could have been effective. However, the accurate evaluation of the clinical evidence of sepsis status and antimicrobial resistance is important before considering any empirical therapy in order to avoid unnecessary treatment which could favor the spread of antimicrobial-resistant strains.

Despite the potential marginal role of the bacteria in the clinical outcome, this study evidenced the presence of multidrug-resistant bacteria in dogs with parvovirosis. In some cases, the isolates showed resistance to the most important antimicrobial drugs in human medicine, such as second- and fourth-generation cephalosporins, macrolides, lincosamides, nitroimidazoles, and glycopeptides while all the Gram-positive bacteria were resistant to fourth-generation cephalosporins and, with the exception of two *Clostridium perfringens* strains, also to nitroimidazoles. A slightly higher sensitivity to the ceftriaxone over cefovecin (third gen. cephalosporins) was also observed.

The analysis of the β-lactamase genes of *Enterobacteriaceae* showed that 15/30 of the strains harbored one to four genes of resistance. In one *E. coli* and one *Klebsiella pneumoniae* from dog id. 22, four genes of resistance (*bla*_TEM_, *bla*_SHV_, *bla*_OXA,_ and *bla*_CTX-M-II_) were detected. Although the gene *bla*_SHV_ is commonly present in most *K. pneumoniae* strains at chromosomal locations, the same combination of different genes present in different bacterial strains in the same dog suggests acquisition through genetic horizontal transfer. Another *K. pneumoniae* derived from dog id. 12 carried the same four genes. The evidence of these bacteria in the enteric tract suggests their potential shedding via feces, and this deserves attention considering their potential zoonotic role and the possibility of spreading to humans. These strains also showed the highest resistance in both the Kirby–Bauer and MIC assays.

In the treatment of dogs with parvoviral enteritis there is no specific therapy, only supportive care approaches. Although some reports warn about the potential risks connected to the use of antibiotics [14,43], an intravenous or subcutaneous broad spectrum of bactericidal antibiotics is commonly used in addition to therapy. Penicillin, alone or in combination with beta-lactamase inhibitors, cephalosporins, fluoroquinolones, metronidazole, and aminoglycosides, is the most commonly used antibiotics that have been reported [15,51,52]. To date, official data on the use or sale of antimicrobials in Italy for the treatment of gastrointestinal infections in dogs are not available. Nonetheless, a guideline for the prudent use of antibiotics in companion animals has been provided [53] along with surveys and cross-sectional studies describing the use of antimicrobials for companion animals [54,55,56]. Similar studies involving other European countries, including Italy, have recently been published [57,58]. Moreover, most recent updates of canine parvoviral enteritis recommend the use of broad-spectrum antibiotics such as ampicillins, cephalosporins, nitroimidazoles, or fluoroquinolones [59]. Although a national survey specifically on the use of antimicrobials in dogs with CPV infection is not available, all of these studies, as well as the guidelines, cite specific antibiotics (i.e., nitroimidazoles such as metronidazole, alone or in addition with spiramycin or cephalosporins) and CIAs (i.e., fluoroquinolones or third-generation cephalosporins) for common use in the generically defined gastrointestinal disease. Since 2017, computerized prescriptions for veterinary medical products (defined as Ricetta Elettronica Veterinaria, REV) have been available in Italy, replacing paper prescriptions for antimicrobials under the direct control of the Ministry of Health. Data analysis could contribute to the categorization of the antimicrobials used for companion animals, supporting future strategies to combat AMR, along with the increasing attention being paid to multi-resistant pathogens found in companion animals that are harmful for humans.

This study outlines the high bacterial resistance to some of the antibiotics commonly used in the treatment of parvoviral enteritis, such as third- and fourth-generation cephalosporins and metronidazole, which pose a high risk of the spread of resistance to antibiotics that are very important for human health. Moreover, inappropriate and ineffective empirical treatments of CPV infection, such as intravenous therapy with narrow-spectrum antimicrobials, potentially contribute to the occurrence of other short- or long-term effects, such as damage to fecal microbiota, neurotoxicity, and chronic gastrointestinal disease [60,61,62]. Given this evidence, the real need for antibiotic therapy and its benefits should be assessed.

The rational rather than empirical use of antibiotics could contribute to the effective control of antimicrobial resistance. The concerns related to AMR are increasing, especially for those involving important antimicrobial classes, such as the third- or higher-generation cephalosporins, glycopeptides, macrolides, ketolides, polymyxins, and quinolones included in the lists of international health institutions [24]. Due to the threat posed to human health, the guidelines on the rational use of antibiotics mainly refer to food-producing animals or animal production practices and the role of companion animals is neglected [63,64]. Moreover, the need to elucidate the role of companion animals in the spread of antibiotic resistance is highlighted by the fact that some of the microorganisms included in the WHO’s list of globally prioritized antibiotic-resistant bacteria [65] are often isolated from companion animals. In our study, four strains of *Klebsiella pneumoniae*, representing a threat to human health in hospital settings [66], showed more than one β-lactamase gene and multidrug resistance. An *E. coli* strain isolated from a young stray dog showed the O111 serogroup and eae genes. The O111 serotype and enteroaggregative intimin (eae) genes related to *E. coli* strains are responsible for diarrhea problems in children [67]. Moreover, four *E. coli*, one *Escherichia fergusonii*, two *Klebsiella pneumoniae,* and one *Enterobacter cloacae* were shown to be resistant to antibiotics considered a last line of defense against resistant infections such as colistin sulphate, imipenem, chloramphenicol, and ceftriaxone. We cannot rule out the possibility that resistant bacterial strains were transferred from humans to animals, since some of the tested puppies might have been abandoned by owners that could not keep the newborn animals. The presence of multidrug resistance could be related to the household environment and it is possible that, for pets with close relations to humans, AMR originates from human sources thus confirming the importance of the One Health approach. Moreover, less common bacteria indicated as potential agents of zoonoses were also isolated: *Salmonella enterica* subsp. *enterica* serovar Schleissheim [68], *E. fergusonii* [69], *Str. pseudoporcinus* [70], and *Str. canis* [71]. This evidence suggests a potential risk for humans connected to the shedding of zoonotic bacteria species carrying drug resistance. Due to this evidence being found in stray and shelter-housed dogs, the roles of these species should be assessed and considered as part of sanitation protocols to limit the contamination of shelters and veterinary clinics, thus limiting the risks posed to the personnel of shelters and veterinarians.

Some limitations—particularly the low availability of tissue samples from dead dogs naturally infected with canine parvovirus—prevent in-depth data analysis. First, the lack of negative controls is a potential limitation: since this was a descriptive study, intended only to evaluate the antimicrobial susceptibility and multidrug-resistance profiles of bacterial species derived from tissue samples of dogs with canine parvovirus infection, samples from CPV-negative dogs were considered non-ideal as negative controls.

As this observational study was based only on samples collected for routine diagnostic purposes, aiming to describe and highlight the presence of multi-resistant bacteria in these targeted individuals, no negative controls were defined.

Another limit was related to the lack of specific anamnestic and clinical information, particularly on the use of antimicrobials for therapies, which prevents speculation on the meaning of the resistance found in the analyzed strains. Therefore, in light of these limitations, further studies are necessary in order to derive in-depth deductions.

Antibiotic treatment is sometimes used in canine parvoviral infection but, as shown in this study, the evidence of multi-resistant bacteria with potential for intra- or cross-species transmission should be carefully considered before unnecessary antimicrobial treatments are undertaken. Dogs, as companion animals, are usually reared and housed in close contact with humans [72,73] and, therefore, a One Health perspective is imperative for global public health.

## 4. Materials and Methods

### 4.1. Clinical Samples

Tissue samples from 23 dead dogs suspected of having parvovirosis were analyzed. Samples were collected from May 2018 to October 2019 and analyzed for diagnostic purposes. Carcasses had already been submitted by public and private veterinary practitioners to ascertain the causa mortis. Most of these subjects were stray dogs (n = 18) and the others were owned dogs (n = 3), shelter dogs (n = 1) and imported dogs (n = 1). The veterinary public services recovered all but one of the roaming strays showing clinical gastroenteric signs, all of which died just after admittance; the other died just before it could be recovered. Other carcasses were submitted by private or public veterinary practitioners with clinical suspicion of infectious gastrointestinal disease in almost all of them. No other anamnestic or clinical information was provided, including vaccination statuses or therapies. The carcasses were subjected to necropsy after admittance or storage at −20 °C. During necropsy, tissue samples (brain, lungs, heart, spleen, liver, intestine, mesenteric lymph nodes, and kidneys) were collected, stored at −20 °C, and subjected to virological and bacteriological assays. The details are summarized in Supplementary Materials Table S1.

### 4.2. Parvovirus PCR and Molecular Characterization of CPV Strains

Organ homogenates were obtained as previously described [74]. DNA was extracted from homogenates using the DNeasy Blood & Tissue Kit (Qiagen S.p.A., Hilden, Germany), according to the manufacturer’s instructions. The presence of CPV DNA was confirmed using a primer pair [75] in a PCR protocol amplifying a 700 bp fragment of the VP2 gene, as previously described [28]. Briefly, PCR was carried out using the GoTaq G2 DNA Polymerase (Promega Italia s.r.l., Milan, Italy) in a 50 μL reaction mix consisting of 10 µL of 5× GoTaq^®^ Reaction Buffer, 1 µL of MgCl_2_ (25 mM), 1 µL of dNTP mix (10 mM), 0.5 µL of each primer VP2-850-Forward and VP2-1550-Reverse (0.5 µM), 0.25 µL of GoTaq^®^ G2 DNA Polymerase, 31.75 μL of nuclease-free water, and 5 μL of DNA extract. Amplification was conducted under the following thermal conditions: 94 °C for 2 min to activate TaqPol followed by 40 cycles of 94 °C for 30 s, 55 °C for 1 min, and 72 °C for 1 min as well as a final extension of 72 °C for 10 min.

The nearly complete VP2 gene sequence (a 1745-bp fragment) was assessed using a primer pair [76] and direct sequencing [77].

Sequencing encompassing both CPV ORFs (including NS and VP genes) was carried out using the primer pairs developed by Pérez et al. [78], as previously described, and the amplicons were directly sequenced using forward, reverse, and internal primers [29].

The nucleotide VP2 coding sequences were obtained using the ClustalW program and analyzed using the BioEdit software. The sequences were submitted to nBLAST to search related sequences in public domain databases. The CPV antigenic variants (CPV-2a, 2b and 2c) were deduced based on the 426-VP2 amino acid residue [79].

To elucidate the genetic relationships between the obtained CPV strains and the dataset of sequences obtained from the NCBI database, a phylogenetic tree was constructed with the MEGA-X software, using the maximum-likelihood (ML) method according to the Tamura 3-parameter (T92) model with discrete Gamma distribution (+G) employing five rate categories, assuming that a certain fraction of the sites were evolutionarily invariable (+I), and employing bootstrap analyses with 1000 replicates. The phylogeny is depicted in Supplementary Materials Figure S2, showing a representative CPV strain for each genetic and antigenic variant.

These sequence data have been previously or newly submitted to the DDBJ/EMBL/GenBank databases under accession numbers reported in Supplementary Materials Table S1.

### 4.3. Additional Virologic Tests

RNA was extracted from samples using the QIAamp Viral RNA Mini Kit (Qiagen S.p.A., Hilden, Germany), according to the manufacturer’s instructions. The extracted DNA and RNA were amplified using a set of gel-based or real-time (RT) PCR assays useful for the detection of CDV [80], CAdVs types 1 and 2 [81], CCoV [82], and CRoV [83]. The details are summarized in Supplementary Materials Table S6.

### 4.4. Bacterial Isolation

For the tissue samples collected from all the dogs, bacterial isolation was performed using selective and differential agar (MacConkey agar, Columbia blood agar and Mannitol Salt agar) incubated at 37 °C for 24 h. Moreover, Columbia blood agar plates were anaerobically incubated with the AnaeroGen™ Anaerobic System (Oxoid, Milano, Italy) to isolate anaerobic bacteria.

For the *Salmonella* spp. culture, pre-enrichment in Buffered Peptone water was performed, followed by two enrichments in Selenite Cystine (SC) and Rappaport-Vassiliadis (RV) broths, and incubated, respectively, at 37 °C and 42 °C for 24 h. The enrichment broths were then plated on Xylose–Lysine Deoxycholate Agar (XLD) and Brilliant Green Agar (BGA).

The identification of the isolated strains was carried out with the biochemical API^®^ system and Vitek^®^ 2 system (bioMérieux, Craponne, France). For the *Salmonella* spp. strains, after identification by API20E^®^, serological typing was performed.

### 4.5. Disk Diffusion Method

The antimicrobial susceptibility of the bacterial strains isolated (n = 43) was evaluated by the disk diffusion method (Kirby-Bauer) on Mueller-Hinton agar, according to the guidelines of the Clinical and Laboratory Standards Institute [84]. A standard panel of 22 antibiotics was used: amoxicillin-clavulanic acid (AMC), 30 μg; ampicillin (AMP), 10 μg; aztreonam (ATM), 30 μg; cefadroxil (CFR), 30 μg; cephalexin (CL), 30 μg; cefovecin (CVN), 30 μg; ceftriaxone (CRO), 30 μg; cefquinome (CEQ), 30 μg; cefuroxime (CXM), 30 μg; clindamycin (DA), 2 μg; chloramphenicol (C), 30 μg; colistin sulfate (CT), 10 μg; doxycycline (DO), 30 μg; enrofloxacin (ENR), 5 μg; gentamicin (CN), 10 μg; imipenem (IPM), 10 μg; marbofloxacin (MAR), 5 μg; methicillin (MET), 5 μg; metronidazole (MPZ), 4 μg; spiramycin (SP), 100 μg; sulfamethoxazole/trimethoprim (SXT), 23.75 μg/1.25 μg; vancomycin (VA), 30 μg. The sensitivity model was evaluated by measuring the diameter of the inhibition zone, and isolates were considered resistant, intermediate, or susceptible according to the CLSI ranges [84].

### 4.6. Determination of Minimum Inhibitory Concentration (MIC)

The minimum inhibitory concentrations (MICs) of 36 of the isolated strains were determined with the Vitek^®^ 2 system (bioMérieux, Craponne, France), with specific panels of antibiotics selected according to the identified species. The VITEK^®^ AST-GN65 card was used to determine the susceptibility of 28 strains of isolated Gram-negative aerobic bacilli, while the VITEK^®^ AST-GP81 card was used to determine the susceptibility of three *Enterococcus* spp. And three Staphylococcus spp. The VITEK^®^ AST-ST03 card was used for the two *Streptococcus* spp., whose MIC values were expressed in µg/mL. According to the breakpoints expressed in vet CLSI 2017 v8.02 and CLSI M100-S25 (2015) [84,85], the isolates were categorized as resistant, intermediate, or susceptible.

### 4.7. Detection of β-Lactamase Genes

Two multiplex PCRs were performed to amplify the β-lactamase genes in the *Enterobacteriaceae* isolates as described by Kim et al. [86]. The first multiplex assay (named Set I) was designed to detect the *bla*_TEM_, *bla*_SHV,_ and *bla*_CTX-M-IV_ group- (8–10) and *bla*_OXA_ β-lactamase-encoding genes, and the second assay (named Set II) was designed to detect *bla*_CTX-M-I_ group-, *bla*_CTX-M-II_ group-, *bla*_CMY-II_-, and *bla*_DHA_-encoding genes. The DNA amplifications were carried out in the GeneAmp™ PCR System 2700 thermal cycle (Applied Biosystems, Foster City, CA, USA). Both assays used identical cycling conditions: the thermal cycling profile consisted of an initial denaturation at 95 °C for 15 min, followed by 30 cycles of 94 °C for 30 s, 61 °C for 90 s, and 72 °C for 90 s and a final extension at 72 °C for 10 min. The sizes of the PCR products were analyzed by electrophoresis on a 2% agarose gel containing GelRed^®^ (Biotium, San Francisco, CA, USA) (4 µL per 100 mL) in 0.5× TBE at 100 V for 1 h, and visualized using GeneSys (Syngene, Cambridge, UK).

### 4.8. Detection of Genes for Toxins of Staphylococcus spp. and of Clostridium perfringens (A to E)

Two multiplex PCR assays were used to amplify the sea-see and tsst-1, eta, etb, mecA (Set I), and seg, seh, sei, sej, and sep (Set II) genes for toxins of *Staphylococcus* spp. as described by Vitale et al. [87].

A multiplex PCR assay was used to detect the toxin genes cpa, cpb, etx, iap, cpe, and cpb2 of *Clostridium perfringens*, according to the method described by Baums et al. [88]. The PCR results were visualized by electrophoresis on a 1.5% agarose gel containing GelRed^®^ (Biotium, San Francisco, CA, USA) (4 µL per 100 mL) in 0.5× TBE at 100 V for 1 h and visualized using GeneSys (Syngene, Cambridge, UK).

### 4.9. Serogroup Identification in E. coli and Virulent Genes’ Identification in Enterobacteriaceae

A multiplex polymerase chain reaction (mPCR) was used to detect the 11 genes that encode serogroup-specific O-antigens and four major virulence factors (eae–intimin adherence protein, enterohemorrhagic hemolysin A (EHEC hlyA), and Shiga toxins [Stx] 1 and 2) so as to detect O157 and the “top six” non-O157 (O26, O45, O103, O111, O121, and O145) Shiga toxin-producing Escherichia coli (STEC) as described by Bai et al. [89]. The search for genes coding for the four virulence factors mentioned above was conducted on all the strains of *Enterobacteriaceae* isolated.

## Figures and Tables

**Table 1:** Bacteria isolated from dogs with canine parvovirus infection.

Species	Family	Order	Number of Isolates	Tissue Sample
Gram-negative				
*Escherichia coli*	*Enterobacteriaceae*	*Enterobacteriales*	19	Brain, heart, intestine, kidney, lymph nodes, liver, spleen
*Klebsiella pneumoniae*			4	Brain, intestine, liver, spleen
*Enterobacter cloacae*			2	Brain, intestine
*Enterobacter gergoviae*			2	Brain, intestine
*Escherichia fergusonii*			1	Intestine
*Proteus mirabilis*			1	Intestine
*Salmonella enterica*			1	Intestine
*Pseudomonas* *aeruginosa*	*Pseudomonadaceae*	*Pseudomonadales*	1	Intestine
Gram-positive				
*Clostridium perfringens*	*Clostridiaceae*	*Clostridiales*	3	Intestine, liver, spleen
*Enterococcus faecium*	*Enterococcaceae*	*Lactobacillales*	2	Brain, lymph nodes
*Enterococcus faecalis*			1	Intestine
*Streptococcus canis*	*Streptococcaceae*	*Lactobacillales*	2	Brain, lung
*Streptococcus* *pseudoporcinus*			1	Intestine
*Staphylococcus lentus*	*Staphylococcaceae*	*Bacillales*	1	Lung
*Staphylococcus sciuri*			1	Brain
*Staphylococcus xylosus*			1	Brain

**Table 2:** Antibiotic sensitivity results derived with the Kirby-Bauer method for the Gram-negative strains (n = 31).

Bacterial Isolates	Dog ID.	AMC	AMP	CFR	CL	CXM	CVN	CRO	CEQ	MET	ATM	IPM	SP	DA	CN	VA	CT	ENR	MAR	MPZ	C	DO	SXT
*Escherichia coli* (n = 19)	1	I	R	R	R	R	R	I	R	R	S	S	R ^a^	R ^a^	R	R ^a^	S	S	S	R	S	I	R
	3	I	R	R	R	R	R	S	R	R	S	I	R ^a^	R ^a^	R	R ^a^	I	S	S	R	S	S	S
	4	I	R	R	R	R	R	R	R	R	R	I	R ^a^	R ^a^	I	R ^a^	I	I	S	R	S	I	R
	5	S	S	R	R	R	I	S	R	R	I	S	R ^a^	R ^a^	I	R ^a^	S	S	S	R	S	S	S
	6	S	S	R	R	R	I	S	R	R	I	S	R ^a^	R ^a^	R	R ^a^	S	S	S	R	S	S	S
	7	S	R	R	R	R	I	S	R	R	S	S	R ^a^	R ^a^	R	R ^a^	S	S	S	R	S	R	R
	8	S	R	R	R	R	I	S	R	R	I	S	R ^a^	R^a^	R	R ^a^	S	S	S	R	S	S	R
	9	I	R	R	R	R	R	S	R	R	I	S	R ^a^	R ^a^	I	R ^a^	S	S	S	R	S	S	S
	10	R	R	R	R	R	R	S	R	R	S	S	R ^a^	R ^a^	R	R ^a^	S	R	R	R	R	R	R
	11	I	R	R	R	R	I	S	R	R	I	S	R ^a^	R ^a^	R	R ^a^	S	R	R	R	S	S	S
	13	S	I	R	R	R	R	S	R	R	R	S	R ^a^	R ^a^	I	R ^a^	I	I	S	R	S	R	S
	14	S	R	S	R	R	S	S	R	R	S	S	R ^a^	R ^a^	R	R ^a^	S	S	S	R	I	R	R
	15	S	S	S	R	R	S	S	R	R	S	I	R ^a^	R ^a^	S	R ^a^	S	S	S	R	S	S	S
	16	S	S	R	S	R	R	S	R	R	R	I	R ^a^	R ^a^	R	R ^a^	S	S	S	R	S	S	S
	17	S	S	S	S	R	S	S	R	R	I	S	R ^a^	R ^a^	I	R ^a^	S	S	S	R	S	S	S
	19	R	R	R	R	R	R	R	R	R	R	S	R ^a^	R ^a^	R	R ^a^	S	R	R	R	R	I	R
	20	S	S	S	S	R	S	S	R	R	S	S	R ^a^	R ^a^	S	R ^a^	S	S	S	R	S	S	S
	22	R	R	R	R	R	I	S	R	R	R	R	R ^a^	R ^a^	R	R ^a^	S	R	R	R	R	I	S
	23	S	S	S	S	R	S	S	R	R	R	S	R ^a^	R ^a^	S	R ^a^	S	I	S	R	S	S	S
*Klebsiella pneumoniae* (n = 4)	6	S	R ^a^	R	R	R	S	S	R	R	S	S	R ^a^	R ^a^	R	R ^a^	S	S	S	R	S	I	R
	10	I	R ^a^	R	R	R	R	S	R	R	S	S	R ^a^	R ^a^	S	R ^a^	S	I	S	R	S	R	S
	12	I	R ^a^	R	R	R	R	R	R	R	R	S	R ^a^	R ^a^	S	R ^a^	S	I	S	R	S	R	R
	22	R	R ^a^	R	R	R	R	R	R	R	R	S	R ^a^	R ^a^	R	R ^a^	S	R	I	R	S	I	R
*Enterobacter cloacae* (n = 2)	2	R	R ^a^	R	R	R	R	R	R	R	R	S	R ^a^	R ^a^	S	R ^a^	S	I	R	R	S	I	R
	13	S	S ^a^	R	R	R	I	S	R	R	I	S	R ^a^	R ^a^	I	R ^a^	S	S	I	R	S	R	S
*Enterobacter gergoviae* (n = 2)	5	S	R ^a^	R	R	R	I	S	R	R	I	S	R ^a^	R ^a^	S	R ^a^	S	I	S	R	S	I	S
	9	S	R ^a^	R	S	R	S	S	R	R	S	S	R ^a^	R ^a^	S	R ^a^	S	S	S	R	S	S	S
*Escherichia fergusoni* (n = 1)	21	S	S	S	S	R	S	S	R	R	S	S	R ^a^	R ^a^	S	R ^a^	R	S	S	R	S	R	S
*Proteus mirabilis* (n = 1)	20	S	S	S	S	R	S	S	R	R	S	S	R ^a^	R ^a^	S	R ^a^	S	S	S	R	S	R ^a^	S
*Salmonella enterica* (n = 1)	3	S	R	R	R	R	I	S	R	R	I	S	R ^a^	R ^a^	R	R ^a^	I	S	S	R	S	S	S
*Pseudomonas aeruginosa* (n = 1)	12	R ^a^	R ^a^	R ^a^	R ^a^	R ^a^	R	S	R	R	R	I	R ^a^	R ^a^	S	R ^a^	S	S	S	R	S ^a^	S ^a^	R

Amoxicillin-clavulanic acid (AMC); ampicillin (AMP); cefadroxil (CFR); cephalexin (CL); cefuroxime (CXM); cefovecin (CVN); ceftriaxone (CRO); cefquinome (CEQ); methicillin (MET); aztreonam (ATM); imipenem (IPM); spiramycin (SP); clindamycin (DA); gentamicin (CN); vancomycin (VA); colistin sulfate (CT); enrofloxacin (ENR); marbofloxacin (MAR); metronidazole (MPZ); chloramphenicol (C); doxycycline (DO); sulfamethoxazole + trimethoprim (SXT). S: sensible; R: resistant; I: intermediate; ^a^ Intrinsic resistance.

**Table 3:** Antibiotic sensitivity results derived with Kirby–Bauer method for the Gram-positive strains (n = 12).

Bacterial Isolates	Dog ID.	AMC	AMP	CFR	CL	CXM	CVN	CRO	CEQ	MET	ATM	IPM	SP	DA	CN	VA	CT	ENR	MAR	MPZ	C	DO	SXT
*Clostridium perfringens* (n = 3)	2	S	R	R	R	R	R	R	R	R	R ^a^	S	R	R	R	S	R ^a^	I	S	R	S	I	R
	3	S	S	S	S	R	I	S	R	S	R ^a^	S	I	S	R	S	R ^a^	S	S	S	S	S	R
	20	S	S	S	S	S	R	S	R	S	R ^a^	S	I	S	R	S	R ^a^	S	S	S	S	S	S
*Enterococcus faecium* (n = 2)	4	I	R	R ^a^	R ^a^	R ^a^	R ^a^	R ^a^	R ^a^	R	R ^a^	R	I ^a^	R	R	S	R ^a^	R	R	R	S	I	I ^a^
	12	I	R	R ^a^	R ^a^	R ^a^	R ^a^	R ^a^	R ^a^	R	R ^a^	R	I ^a^	I	R	S	R ^a^	R	R	R	S	I	R ^a^
*Enterococcus faecalis* (n = 1)	9	S	S	S ^a^	R ^a^	R ^a^	R ^a^	I^a^	R ^a^	R	R ^a^	S	R ^a^	R ^a^	S	S	R ^a^	R	R	R	S	R	R ^a^
*Staphylococcus lentus* (n = 1)	20	S	S	S	S	S	S	S	R	S ^a^	R ^a^	S	I	I	S	S	R ^a^	S	S	R	S	S	S
*Staphylococcus sciuri* (n = 1)	10	S	S	S	S	R	S	S	R	S ^a^	R ^a^	S	I	I	S	S	R ^a^	S	S	R	S	S	S
*Staphylococcus xylosus* (n = 1)	4	S	R	R	R	R	R	I	R	R ^a^	R ^a^	S	R	R	S	S	R ^a^	R	S	R	S	S	S
*Streptococcus canis* (n = 2)	1	S	I	I	S	S	S	S	R	S	R ^a^	S	I	R	R	S	R ^a^	I	S	R	S	S	S
	5	S	S	S	S	R	R	S	R	S	R ^a^	S	I	S	I	S	R ^a^	I	S	R	S	R	S
*Streptococcus pseudoporcinus* (n = 1)	18	S	S	S	S	R	I	S	R	S	R ^a^	S	I	I	I	S	R ^a^	I	I	R	S	S	S

Amoxicillin-clavulanic acid (AMC); ampicillin (AMP); cefadroxil (CFR); cephalexin (CL); cefuroxime (CXM); cefovecin (CVN); ceftriaxone (CRO); cefquinome (CEQ); methicillin (MET); aztreonam (ATM); imipenem (IPM); spiramycin (SP); clindamycin (DA); gentamicin (CN); vancomycin (VA); colistin sulfate (CT); enrofloxacin (ENR); marbofloxacin (MAR); metronidazole (MPZ); chloramphenicol (C); doxycycline (DO); sulfamethoxazole + trimethoprim (SXT). S: sensible; R: resistant; I: intermediate; ^a^ Intrinsic resistance.

**Table 4:** β-lactamase genes, and antibiotic sensitivity results derived with the Kirby–Bauer method for the *Enterobacteriaceae* isolates.

Dog ID.	Bacterial Isolates	Source	β-Lactamase Genes	AMC	AMP	CFR	CL	CXM	CVN	CRO	CEQ	MET	ATM	IPM
1	*E. coli*	Intestine	*bla*_TEM_, *bla*_CMY-II_, *bla*_CTX-M-I_	I	R	R	R	R	R	I	R	R	S	S
2	*E. cloacae*	Intestine	*bla*_TEM_, *bla*_DHA_	R	R ^a^	R	R	R	R	R	R	R	R	S
3	*S. enterica*	Intestine	negative	S	R	R	R	R	I	S	R	R	I	S
	*E. coli*	Intestine	negative	I	R	R	R	R	R	S	R	R	S	I
4	*E. coli*	Intestine	*bla*_TEM_, *bla*_CTX-M-II_	I	R	R	R	R	R	R	R	R	R	I
5	*E. gergoviae*	Brain	negative	S	R^a^	R	R	R	I	S	R	R	I	S
	*E. coli*	Intestine	negative	S	S	R	R	R	I	S	R	R	I	S
6	*E. coli*	Intestine	negative	S	S	R	R	R	I	S	R	R	I	S
	*K. pneumoniae*	Brain	*bla*_TEM_, *bla*_SHV_, *bla*_CTX-M-II_	S	R ^a^	R	R	R	S	S	R	R	S	S
7	*E. coli*	Intestine	negative	S	R	R	R	R	I	S	R	R	S	S
8	*E. coli*	Intestine	*bla*_TEM_	S	R	R	R	R	I	S	R	R	I	S
9	*E. coli* (0111, *eae*)	Intestine	*bla*_TEM_, *bla*_CTX-M-II_	I	R	R	R	R	R	S	R	R	I	S
	*E. gergoviae*	Intestine	*bla*_SHV_	S	R ^a^	R	S	R	S	S	R	R	S	S
10	*E. coli*	Intestine	negative	R	R	R	R	R	R	S	R	R	S	S
	*K. pneumoniae*	Brain	*bla*_SHV_	I	R ^a^	R	R	R	R	S	R	R	S	S
11	*E. coli*	Intestine	*bla*_TEM_, *bla*_OXA_	I	R	R	R	R	I	S	R	R	I	S
12	*K. pneumoniae*	Intestine	*bla*_TEM_, *bla*_OXA_, *bla*_SHV_*bla*_CTX-M-II_	I	R ^a^	R	R	R	R	R	R	R	R	S
13	*E. coli*	Intestine	negative	S	I	R	R	R	R	S	R	R	R	S
	*E. cloacae*	Brain	negative	S	S ^a^	R	R	R	I	S	R	R	I	S
14	*E. coli*	Intestine	*bla*_TEM_, *bla*_CTX-M-II_	S	R	S	R	R	S	S	R	R	S	S
15	*E. coli*	Intestine	negative	S	S	S	R	R	S	S	R	R	S	I
16	*E. coli*	Intestine	negative	S	S	R	S	R	R	S	R	R	R	I
17	*E. coli*	Intestine	negative	S	S	S	S	R	S	S	R	R	I	S
19	*E. coli*	Intestine	*bla*_OXA_, *bla*_CTX-M-II_	R	R	R	R	R	R	R	R	R	R	S
20	*E. coli*	Intestine	negative	S	S	S	S	R	S	S	R	R	S	S
	*P. mirabilis*	Intestine	negative	S	S	S	S	R	S	S	R	R	S	S
21	*E. fergusonii*	Intestine	*bla*_CTX-M-II_	S	S	S	S	R	S	S	R	R	S	S
22	*E. coli*	Intestine	*bla*_TEM_, *bla*_OXA_, *bla*_SHV_, *bla*_CTX-M-II_	R	R	R	R	R	I	S	R	R	R	R
	*K. pneumoniae*	Intestine	*bla*_TEM_, *bla*_OXA_, *bla*_SHV_, *bla*_CTX-M-II_	R	R ^a^	R	R	R	R	R	R	R	R	S
23	*E. coli*	Intestine	negative	S	S	S	S	R	S	S	R	R	R	S

Amoxicillin–clavulanic acid (AMC); ampicillin (AMP); cefadroxil (CFR); cephalexin (CL); cefuroxime (CXM); cefovecin (CVN); ceftriaxone (CRO); cefquinome (CEQ); methicillin (MET); aztreonam (ATM); imipenem (IPM); ^a^ Intrinsic resistance.

**Table 5:** Multidrug-resistance evaluation of 27 strains of the *Enterobacteriaceae* family.

Bacterial Isolates	Dog Id	Penicillins				Cephalosporins				Carbapenem		Aminoglycosides		Fluoroquinolones				Chloramphenicol		Sulfonamides		Beta Lactams		Tetracycline	
		AMC		AMP		CL		CVN		IPM		CN		ENR		MAR		C		SXT		MET	PIP	DO	TE
		KB	MIC	KB	MIC	KB	MIC	KB	MIC	KB	MIC	KB	MIC	KB	MIC	KB	MIC	KB	MIC	KB	MIC	KB	MIC	KB	MIC
*E. coli* (n = 16)	1	I	R	R	R	R	R	R	R	S	S	R	S	S	S	S	S	S	S	R	S	R	R	I	R
	3	I	S	R	S	R	R	R	S	I	S	R	R	S	S	S	S	S	S	S	S	R	S	S	S
	4	I	R	R	R	R	R	R	R	I	S	I	R	I	S	S	S	S	S	R	R	R	R	I	R
	5	S	S	S	S	R	R	I	S	S	S	I	R	S	S	S	S	S	S	S	S	R	S	S	S
	6	S	S	S	S	R	R	I	S	S	S	R	R	S	S	S	S	S	S	S	S	R	S	S	S
	7	S	S	R	R	R	R	I	S	S	S	R	S	S	S	S	S	S	S	R	R	R	R	R	R
	8	S	S	R	R	R	R	I	S	S	S	R	R	S	S	S	S	S	S	R	R	R	R	S	S
	9	I	S	R	R	R	R	R	S	S	S	I	R	S	S	S	S	S	S	S	S	R	R	S	S
	10	R	R	R	R	R	R	R	S	S	S	R	S	R	R	R	R	R	R	R	R	R	R	R	R
	11	I	R	R	R	R	R	I	S	S	S	R	R	R	R	R	R	S	S	S	S	R	R	S	S
	13	S	S	I	S	R	R	R	S	S	S	I	R	I	S	S	S	S	S	S	S	R	R	R	S
	14	S	S	R	R	R	R	S	S	S	S	R	S	S	S	S	S	I	I	R	R	R	S	R	R
	15	S	S	S	S	R	R	S	S	I	S	S	R	S	S	S	S	S	I	S	S	R	R	S	S
	16	S	S	S	S	S	R	R	S	I	S	R	R	S	S	S	S	S	S	S	S	R	S	S	S
	17	S	S	S	S	S	R	S	S	S	S	I	R	S	S	S	S	S	S	S	S	R	S	S	S
	19	R	R	R	R	R	R	R	S	S	S	R	R	R	S	R	R	R	S	R	S	R	S	I	S
	20	R	S	R	R	R	S	I	S	R	S	R	S	R	R	R	R	R	R	S	S	R	S	I	R
*Klebsiella pneumoniae* (n = 4)	6	S	S	R ^a^	R ^a^	R	S	S	S	S	S	R	R	S	S	S	S	S	S	R	R	R	R	I	R
	10	S	I	R ^a^	R ^a^	R	S	R	S	S	S	S	S	I	S	S	S	S	S	S	S	R	R	R	R
	12	I	I	R ^a^	R ^a^	R	R	R	R	S	S	S	S	I	I	S	S	S	S	R	R	R	R	R	R
	20	R	I	R ^a^	R ^a^	R	R	R	R	S	S	R	R	R	R	I	I	S	S	R	R	R	R	I	R
*Enterobacter cloacae* (n = 2)	2	R	R	R ^a^	nd	R	R	R	S	S	S	S	S	I	I	R	S	S	S	R	R	R	R	I	R
	13	S	R	S ^a^	nd	R	R	I	S	S	S	I	S	S	S	I	S	S	S	S	S	R	S	R	R
*Enterobacter gergoviae* (n = 2)	5	S	R	R ^a^	nd	R	R	I	S	S	S	S	S	I	S	S	S	S	S	S	S	R	S	I	S
	9	S	R	R ^a^	nd	S	R	S	S	S	S	S	S	S	S	S	S	S	S	S	S	R	I	S	S
*Escherichia fergusoni* (n = 1)	23	S	S	S	S	S	S	S	S	S	S	S	S	S	S	S	S	S	S	S	S	R	S	R	S
*Proteus mirabilis* (n = 1)	22	S	S	S	S	S	I	S	S	S	S	S	S	S	S	S	S	S	S	S	S	R	S	R ^a^	R
*Salmonella enterica* (n = 1)	3	S	S	R	S	R	S	I	S	S	S	R	S	S	S	S	S	S	S	S	S	R	S	S	S

Amoxicillin + clavulanic acid (AMC); ampicillin (AMP); cephalexin (CL); cefovecin (CVN); imipenem (IPM); gentamicin (CN); enrofloxacin (ENR); marbofloxacin (MAR); chloramphenicol (C); sulfamethoxazole + trimethoprim (SXT); methicillin (MET); piperacillin (PIP); doxycycline (DO); tetracycline (TE); ^a^ Intrinsic resistance; nd: not determined.

## Data Availability

Sequence data were submitted to the DDBJ/EMBL/GenBank databases under accession numbers MT981020–MT981039.

## Supplementary Materials

The following are available online at https://www.mdpi.com/article/10.3390/antibiotics11020142/s1. Figure S1: Gross lesions observed at necropsy, Figure S2: Maximum likelihood tree based on 158 full-length VP2 gene sequences of canine parvovirus type 2 strains, Table S1: Details of collected and tested samples, Table S2: Antibiotic sensitivity results according to minimum inhibitory concentration (MIC) method for the Gram-negative strains (n = 28), Table S3: Antibiotic sensitivity results with minimum inhibitory concentration (MIC) method for the Gram-positive strains (n = 8), Table S4: Comparison of the results obtained with the two methods for MDR Enterobacteriaceae strains (n = 15), Table S5: Comparison of the results obtained with the two methods for MDR Gram-positive strains (n = 2), Table S6: Details on additional virologic tests.
