# Supplementary material for: Antimicrobial Resistance (AMR) of Bacteria Isolated from Dogs with Canine Parvovirus (CPV) Infection: The Need for a Rational Use of Antibiotics in Companion Animal Health
Source: Antibiotics (Basel). 2022 Jan 23;11(2):142. doi: 10.3390/antibiotics11020142 (PMC8868125; doi:10.3390/antibiotics11020142)
Supplement: Supplementary file 1 [file antibiotics-11-00142-s001.zip › antibiotics-1500206-supplementary/Supplementary Material - Figure S1.pdf]

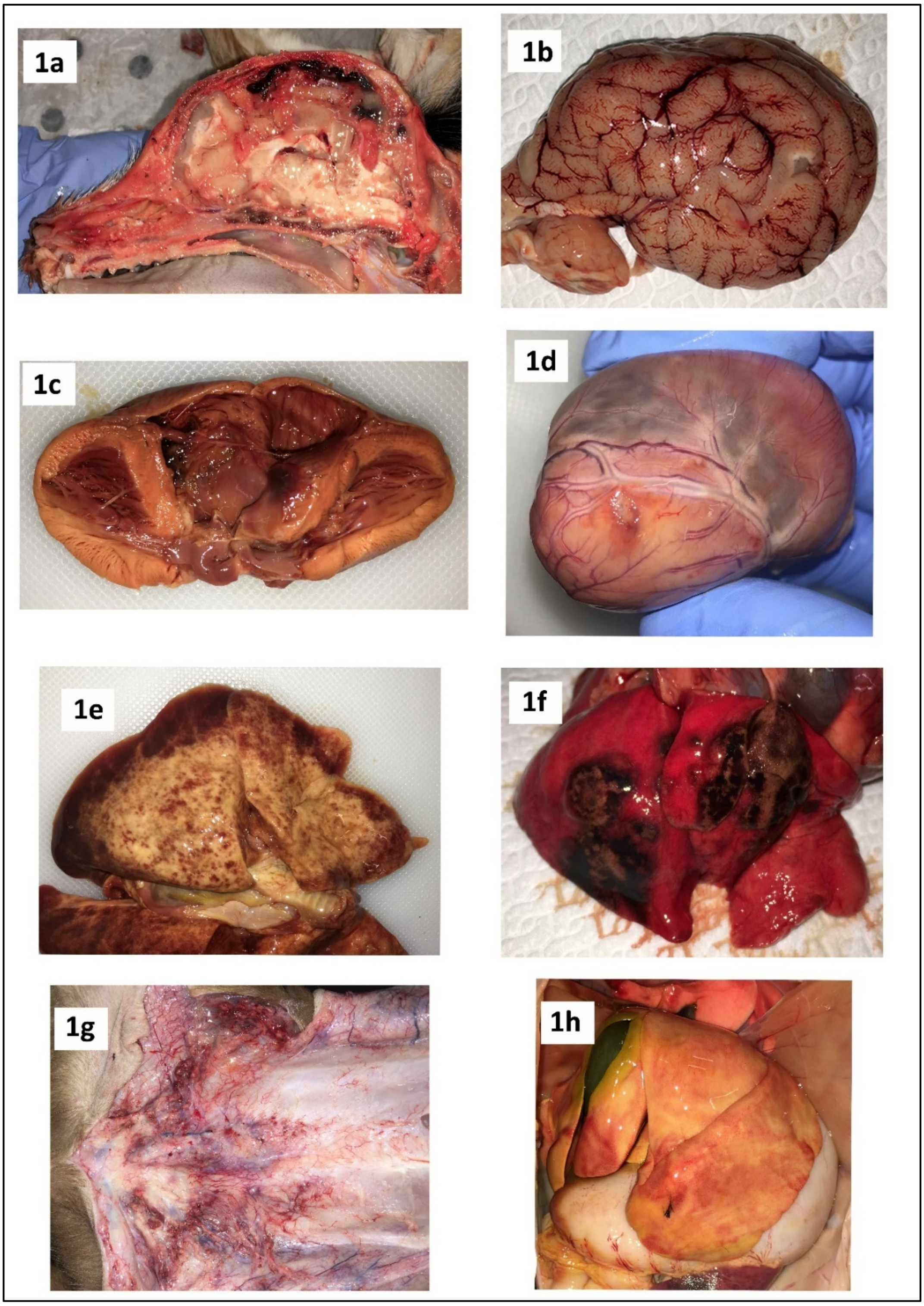

Supplementary Material Figure S1: Gross lesions observed at necropsy: brain oedema and haemorrhage (1a, 1b); paleness and focal fibrous lesions of myocardium (1b); ecchymoses, petechiae and necrosis on lungs (1c), subcutaneous petechiae (1d), and icterus and hepatic lipidosis (1e).
