# Supplementary material for: Antimicrobial Resistance (AMR) of Bacteria Isolated from Dogs with Canine Parvovirus (CPV) Infection: The Need for a Rational Use of Antibiotics in Companion Animal Health
Source: Antibiotics (Basel). 2022 Jan 23;11(2):142. doi: 10.3390/antibiotics11020142 (PMC8868125; doi:10.3390/antibiotics11020142)
Supplement: Supplementary file 1 [file antibiotics-11-00142-s001.zip › antibiotics-1500206-supplementary/Supplementary Material - Figure S2.pdf]

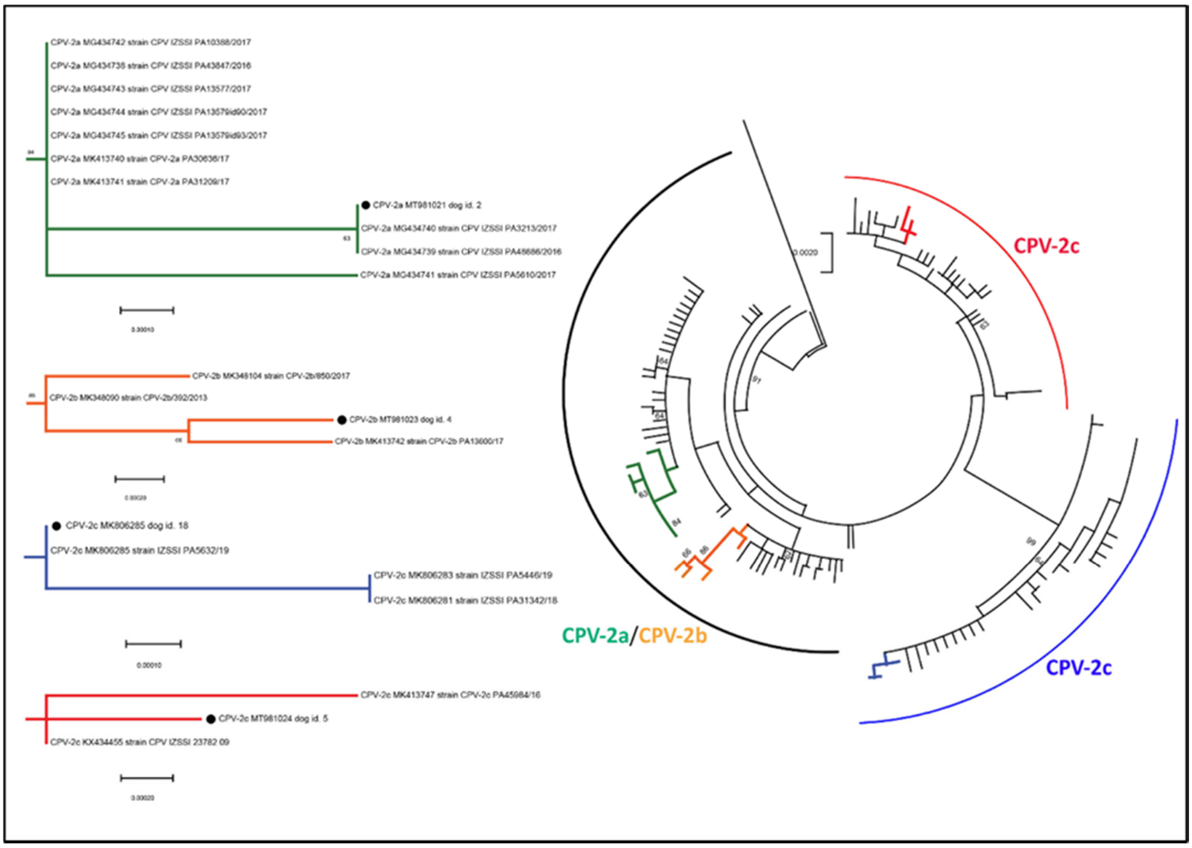


**Supplementary Material Figure S2:** Maximum likelihood tree (on the right) based on 158 full length VP2 gene sequences of canine parvovirus type 2 strains (bootstrap 1,000 replicates; bootstrap values greater than 60 are shown). CPV-2a/2b strain sequences (black arch). CPV-2c strain sequences related to Asian CPV-2c strains (blue arch). CPV-2c strain sequences related to European/South American CPV-2c strains (red arch). Separate branches (on the left) indicate the sub-clusters of CPV - 2a (green), CPV-2b (orange), CPV - 2c (blue and red) strains from Italy analysed in this study. Black dots markings (●) indicate the CPV strains representative of each genetic/antigenic variant analysed in this study. Each sequence is indicated with virus variant (CPV - 2a, CPV - 2b, CPV - 2c), accession number and strain name/dog id. The scale bar indicates the estimated numbers of nucleotide substitutions per site.
