# Supplementary material for: Antimicrobial Resistance (AMR) of Bacteria Isolated from Dogs with Canine Parvovirus (CPV) Infection: The Need for a Rational Use of Antibiotics in Companion Animal Health
Source: Antibiotics (Basel). 2022 Jan 23;11(2):142. doi: 10.3390/antibiotics11020142 (PMC8868125; doi:10.3390/antibiotics11020142)
Supplement: Supplementary file 1 [file antibiotics-11-00142-s001.zip › antibiotics-1500206-supplementary/Supplementary Material - Table S1.pdf]

Supplementary Material - Table S1. Details on collected and tested samples.

Nr.	Date of sampling	Breed	Origin	Age	Collected samples	CPV variant	GenBank Accession number	Reference	Viral co-infections
1	23-May-2018	Cane corso	Owned dog	80 days	Intestine, liver, lung, kidney, spleen, heart	CPV-2a	MT981020	This study	no
2	04-Jun-2018	Mixed breed	Stray dog	2 months	Brain, heart, liver, intestine, mesenteric lymph nodes, spleen, lung, kidney, spinal cord	CPV-2a	MT981021	This study	no
3	25-Jun-2018	Mixed breed	Stray dog	1 year	Brain, heart, liver, intestine, mesenteric lymph nodes, spleen, lung, kidney, uterus, spinal cord	CPV-2a	MT981022	This study	no
4	09-Jul-2018	Mixed breed	Stray dog	40 days	Brain, heart, liver, intestine, mesenteric lymph nodes, lung, kidney	CPV-2b	MT981023	This study	no
5	28-Aug-2018	Rottweiler	Stray dog	10 months	Brain, heart, liver, intestine, mesenteric lymph nodes, spleen, lung, kidney, thymus, spinal cord, bone marrow	CPV-2c	MT981024	This study	no
6	07-Sep-2018	Mixed breed	Stray dog	50 days	Brain, heart, liver, intestine, mesenteric lymph nodes, spleen, lung, kidney	CPV-2c	MT981025	This study	CCoV[a]
7	07-Sep-2018	Mixed breed	Stray dog	3 months	Brain, heart, liver, intestine, mesenteric lymph nodes, spleen, lung, kidney	CPV-2c	MT981026	This study	CCoV[a]
8	11-Sep-2018	Mixed breed	Stray dog	2 months	Brain, heart, liver, intestine, mesenteric lymph nodes, spleen, lung, kidney	CPV-2c	MK806279	[31]	no
9	11-Sep-2018	Mixed breed	Stray dog	2 months	Brain, heart, liver, intestine, mesenteric lymph nodes, spleen, lung, kidney	CPV- 2c	MK806280	[31]	no
10	11-Sep-2018	Mixed breed	Stray dog	2 months	Brain, heart, liver, intestine, mesenteric lymph nodes, spleen, lung, kidney	CPV-2b	MT981027	This study	no
11	12-Sep-2018	Mixed breed	Owned dog	3 months	Brain, heart, liver, intestine, mesenteric lymph nodes, spleen, lung, kidney	CPV-2b	MT981028	This study	CCoV[a]
12	24-Oct-2018	Mixed breed	Owned dog	50 days	Brain, heart, liver, intestine, mediastinal and mesenteric	CPV-2a	MT981029	This study	no

					lymph nodes, spleen, lung, kidney, skin				
13	12-Nov-2018	Mixed breed	Shelter dog	4 months	Brain, heart, liver, intestine, mesenteric lymph nodes, spleen, lung, kidney	CPV-2a	MT981030	This study	no
14	06-Feb-2019	Pitt bull	Stray dog	13 months	Heart, liver, intestine, mesenteric lymph nodes, spleen, lung, kidney	CPV-2a	MT981031	This study	no
15	21-Feb-2019	Mixed breed	Stray dog	20 months	Brain, heart, liver, intestine, mesenteric lymph nodes, spleen, lung, kidney	CPV-2c	MT981032	This study	no
16	21-Feb-2019	Mixed breed	Stray dog	4 months	Brain, heart, liver, intestine, mesenteric lymph nodes, spleen, lung, kidney	CPV-2c	MT981033	This study	no
17	21-Feb-2019	Mixed breed	Stray dog	2 years	Brain, heart, liver, intestine, mesenteric lymph nodes, spleen, lung, kidney	CPV-2a	MT981034	This study	no
18	04-Mar-2019	Mixed breed	Stray dog	2 years	Heart, liver, intestine, spleen, lung, kidney	CPV- 2c	MK806285	[31]	no
19	05-May-2019	Mixed breed	Stray dog	2 years	Brain, heart, liver, intestine, spleen, lung, kidney	CPV- 2c	MT981035	This study	CAdV-1[b] CCoV[a]
20	30-Sep-2019	Mixed breed	Stray dog	4 months	Heart, intestine, spleen, lung, kidney	CPV- 2c	MT981036	This study	no
21	30-Sep-2019	Neapolitan mastiff	Imported dog	9 months	Heart, liver, intestine, spleen, lung, kidney	CPV-2a	MT981037	This study	CCoV[a]
22	03-Oct-2019	Mixed breed	Stray dog	5 months	Brain, heart, liver, intestine, mesenteric lymph nodes, spleen, lung, kidney	CPV-2a	MT981038	This study	CCoV[a]
23	16-Oct-2019	Mixed breed	Stray dog	2 years	Heart, liver, intestine, spleen, lung, kidney	CPV-2a	MT981039	This study	no

[a]CCoV: canine coronavirus; [b]CAdV-1: canine adenovirus type 1.
