# Supplementary material for: Antimicrobial Resistance (AMR) of Bacteria Isolated from Dogs with Canine Parvovirus (CPV) Infection: The Need for a Rational Use of Antibiotics in Companion Animal Health
Source: Antibiotics (Basel). 2022 Jan 23;11(2):142. doi: 10.3390/antibiotics11020142 (PMC8868125; doi:10.3390/antibiotics11020142)
Supplement: Supplementary file 1 [file antibiotics-11-00142-s001.zip › antibiotics-1500206-supplementary/Supplementary Material - Table S2.pdf]

Supplementary Material - Table S2. Antibiotic sensitivity results with minimum inhibitory concentration (MIC) method for the Gram-negative strains (n=28)

Bacterial isolates	Dog id	AMC	AMP	CL	CVN	CPD	FUR	PIP	IPM	AK	CN	TM	ENR	MAR	C	TE	SXT	FT
E. coli *(n=16)*	1	**≥32**	**≥32**	**≥64**	**≥8**	**≥8**	**≥8**	**≥128**	≤1	≤2	**≥16**	2	≤0,12	≤0,5	4	**≥16**	≤20	≤16
	3	4	8	**8**	≤0,5	≤0,25	≤1	≤4	≤1	≤2	≤1	≤1	≤0,12	≤0,5	4	≤1	≤20	≤16
	4	**16**	**≥32**	**≥64**	**≥8**	**≥8**	**≥8**	**≥128**	≤1	≤2	≤1	≤1	≤0,12	≤0,5	4	**≥16**	**≥320**	≤16
	5	≤2	≤2	**8**	≤0,5	≤0,25	≤1	≤4	≤1	≤2	≤1	≤1	≤0,12	≤0,5	4	≤1	≤20	≤16
	6	4	4	**8**	1	0,5	≤1	≤4	≤1	≤2	≤1	≤1	≤0,12	≤0,5	8	≤1	≤20	≤16
	7	8	**≥32**	**8**	≤0,5	≤0,25	≤1	**≥128**	≤1	≤2	**≥16**	4	≤0,12	≤0,5	8	**≥16**	**≥320**	≤16
	8	4	**≥32**	**8**	≤0,5	≤0,25	≤1	**≥128**	≤1	≤2	≤1	≤1	≤0,12	≤0,5	≤2	≤1	**≥320**	≤16
	9	8	**≥32**	**8**	≤0,5	≤0,25	≤1	**≥128**	≤1	≤2	≤1	≤1	≤0,12	≤0,5	4	≤1	≤20	≤16
	10	**16**	**≥32**	**8**	1	≤0,25	≤1	**≥128**	≤1	≤2	**≥16**	8	**≥4**	**≥4**	**≥64**	**≥16**	**≥320**	≤16
	11	**16**	**≥32**	**8**	1	0,5	≤1	**≥128**	≤1	≤2	≤1	**≥16**	**≥4**	**≥4**	4	≤1	≤20	≤16
	13	4	8	**8**	1	≤0,25	≤1	≤4	≤1	≤2	≤1	≤1	≤0,12	≤0,5	4	≤1	≤20	≤16
	14	8	**≥32**	**8**	≤0,5	≤0,25	≤1	**≥128**	≤1	≤2	**≥16**	4	≤0,12	≤0,5	16	**≥16**	**≥320**	≤16
	15	≤2	8	**8**	≤0,5	0,5	≤1	≤4	≤1	≤2	≤1	≤1	≤0,12	≤0,5	16	≤1	≤20	≤16
	16	≤2	8	**8**	1	0,5	≤1	≤4	≤1	≤2	≤1	≤1	≤0,12	≤0,5	8	≤1	≤20	≤16
	17	≤2	4	**8**	≤0,5	0,5	≤1	≤4	≤1	≤2	≤1	≤1	≤0,12	≤0,5	8	≤1	≤20	≤16
	22	**16**	**≥32**	≤4	1	0,5	≤1	32	≤1	4	**≥16**	**≥16**	**≥4**	**≥4**	**≥64**	**≥16**	≤20	≤16
Klebsiella pneumoniae *(n=4)*	6	8	**≥32**[a]	≤4	≤0,5	≤0,25	≤1	**≥128**	≤1	≤2	**≥16**	8	≤0,12	≤0,5	4	**≥16**	**≥320**	≤16
	10	4	**≥32**[a]	≤4	1	≤0,25	≤1	**≥128**	≤1	≤2	≤1	≤1	≤0,12	≤0,5	≤2	**≥16**	≤20	≤16
	12	16	**≥32**[a]	**≥64**	**≥8**	**≥8**	**≥8**	**≥128**	≤1	≤2	≤1	8	1	≤0,5	≤2	**≥16**	**≥320**	32
	22	16	**≥32**[a]	**≥64**	**≥8**	**≥8**	**≥8**	**≥128**	≤1	≤2	**≥16**	**≥16**	**≥4**	2	≤2	**≥16**	**≥320**	≤16
Enterobacter cloacae *(n=2)*	2	**≥32**	nd	**≥64**	**≥8**	**≥8**	**≥8**	**≥128**	≤1	≤2	≤1	≤1	1	≤0,5	4	**≥16**	**≥320**	≤16
	13	**≤2**	nd	**8**	1	≤0,25	≤1	≤4	≤1	≤2	≤1	≤1	≤0,12	≤0,5	4	**≥16**	≤20	≤16
Enterobacter gergoviae *(n=2)*	5	**4**	nd	**≤4**	1	≤0,25	≤1	8	≤1	≤2	≤1	≤1	≤0,12	≤0,5	≤2	≤1	≤20	≤16
	9	**≤2**	nd	**≤4**	≤0,5	≤0,25	≤1	32	≤1	≤2	≤1	≤1	≤0,12	≤0,5	≤2	≤1	≤20	32
Escherichia fergusoni *(n=1)*	21	≤2	≤2	8	≤0,5	1	≤1	≤4	≤1	≤2	≤1	≤1	≤0,12	≤0,5	4	≤1	≤20	≤16
Proteus mirabilis *(n=1)*	20	≤2	≤2	16	≤0,5	≤0,25	≤1	≤4	nd	≤2	≤1	≤1	≤0,12	≤0,5	4	**≥16**	≤20	**≥64**
Salmonella enterica *(n=1)*	3	≤2	≤2	8	≤0,5	≤0,25	2	≤4	≤1	≤2	≤1	≤1	≤0,12	≤0,5	8	≤1	≤20	32
Pseudomonas aeruginosa *(n=1)*	12	nd	nd	nd	nd	nd	nd	8	2	≤2	≤1	≤1	0,5	≤0,5	nd	nd	nd	nd

Amoxicillin-clavulanic acid (AMC); ampicillin (AMP); cefalexin (CL); cefovecin (CVN); cefpodoxime (CPD); ceftiofur (FUR); piperacillin (PIP); imipenem (IPM); amikacina (AK); gentamicin (CN); tobramycin (TM); enrofloxacin (ENR); marbofloxacin (MAR); chloramphenicol (C); tetracycline (TE); sulfamethoxazole + trimethoprim (SXT); nitrofurantoin (FT); [a]Intrinsic resistance [32]; nd: not determined.
