# Supplementary material for: Antimicrobial Resistance (AMR) of Bacteria Isolated from Dogs with Canine Parvovirus (CPV) Infection: The Need for a Rational Use of Antibiotics in Companion Animal Health
Source: Antibiotics (Basel). 2022 Jan 23;11(2):142. doi: 10.3390/antibiotics11020142 (PMC8868125; doi:10.3390/antibiotics11020142)
Supplement: Supplementary file 1 [file antibiotics-11-00142-s001.zip › antibiotics-1500206-supplementary/Supplementary Material - Table S3.pdf]

Supplementary Material - Table S3. Antibiotic sensitivity results with minimum inhibitory concentration (MIC) method for the Gram-positive strains (n=8).

Bacterial isolates	Dog id	AMP	BNZ	CRO	CTX	E	DA	AK	CN	VA	ENR	LVX	MAR	MXF	PRD	C	F	DO	MNO	TE	SXT	LZD	FT	TG
Enterococcus faecium (n=2)	4	nd	nd	nd	nd	4	nd	nd	nd	nd	**≥4**	nd	**≥4**	nd	nd	≤4	≤4	**≥16**	nd	nd	nd	nd	32	nd
	12	nd	nd	nd	nd	**≥8**	nd	nd	nd	nd	**≥4**	nd	**≥4**	nd	nd	8	≤4	**≥16**	nd	nd	nd	nd	64	nd
Enterococcus faecalis (n=1)	9	nd	nd	nd	nd	4	nd	nd	nd	nd	≤0,5	nd	1	nd	nd	≤4	≤4	**≥16**	nd	nd	nd	nd	≤16	nd
Staphylococcus lentus (n=1)	20	nd	nd	nd	nd	0,5	0,25	≤2	≤0,5	nd	≤0,5	nd	1	nd	0,25	≤4	≤4	≤0,5	≤0,5	nd	≤10	nd	≤16	nd
Staphylococcus sciuri (n=1)	10	nd	nd	nd	nd	≤0,25	0,5	≤2	≤0,5	nd	≤0,5	nd	1	nd	0,25	≤4	≤4	≤0,5	≤0,5	nd	≤10	nd	≤16	nd
Staphylococcus xylosus (n=1)	4	nd	nd	nd	nd	1	**0,25**	≤2	≤0,5	nd	**≥4**	nd	**≥4**	nd	1	≤4	≤4	**≥16**	**≥16**	nd	≤10	nd	32	nd
Streptococcus canis (n=1)	5	≤0,25	≤0,06	≤0,12	≤0,12	≤0,12	≤0,25	nd	nd	≤0,12	nd	≤0,5	nd	≤0,12	nd	≤1	nd	nd	nd	**≥16**	≤10	≤2	nd	0,12
Streptococcus pseudoporcinus (n=1)	18	≤0,25	≤0,06	≤0,12	≤0,12	≤0,12	≤0,25	nd	nd	≤0,12	nd	≤0,5	nd	≤0,12	nd	2	nd	nd	nd	1	≤10	≤2	nd	≤0,06

Ampicillin (AMP); benzilpenicillina (BNZ); ceftriaxone (CRO); cefotaxime (CTX); erythromycin (E); clindamycin (DA); amikacin (AK); gentamicin (CN); vancomycin (VA); enrofloxacin (ENR); levofloxacin (LVX); marbofloxacin (MAR); moxifloxacin (MXF); pradofloxacin (PRD); chloramphenicol (C); florphenicol (F); doxycycline (DO); minocycline (MNO); tetracyclines (TE); sulfamethoxazole + trimethoprim (SXT); linezolid (LZD); nitrofurantoin (FT); tigecycline (TG); nd: not determined.
