# Supplementary material for: Antimicrobial Resistance (AMR) of Bacteria Isolated from Dogs with Canine Parvovirus (CPV) Infection: The Need for a Rational Use of Antibiotics in Companion Animal Health
Source: Antibiotics (Basel). 2022 Jan 23;11(2):142. doi: 10.3390/antibiotics11020142 (PMC8868125; doi:10.3390/antibiotics11020142)
Supplement: Supplementary file 1 [file antibiotics-11-00142-s001.zip › antibiotics-1500206-supplementary/Supplementary Material - Table S4.pdf]

Supplementary Material - Table S4. Comparison of the results obtained with the two methods for MDR *Enterobacteriaceae* strains (n=15).

		Penicillins				Cephalosporins				Carbapenem		Aminoglycosides		Fluoroquinolones				Chloramphenicol		Sulfonamides		Beta lactams		Tetracyclines	
**Bacterial isolates**	**Dog id.**	**AMC**		**AMP**		**CL**		**CVN**		**IPM**		**CN**		**ENR**		**MAR**		**C**		**SXT**		**MET**	**PIP**	**DO**	**TE**
		KB	MIC	KB	MIC	KB	MIC	KB	MIC	KB	MIC	KB	MIC	KB	MIC	KB	MIC	KB	MIC	KB	MIC	KB	MIC	KB	MIC
Escherichia coli (n=10)	1	I	**R**	**R**	**R**	**R**	**R**	**R**	**R**	S	S	**R**	S	S	S	S	S	S	S	**R**	S	**R**	**R**	I	**R**
	4	I	**R**	**R**	**R**	**R**	**R**	**R**	**R**	I	S	I	**R**	I	S	S	S	S	S	**R**	**R**	**R**	**R**	I	**R**
	7	S	S	**R**	**R**	**R**	**R**	I	S	S	S	**R**	S	S	S	S	S	S	S	**R**	**R**	**R**	**R**	**R**	**R**
	8	S	S	**R**	**R**	**R**	**R**	I	S	S	S	**R**	**R**	S	S	S	S	S	S	R	**R**	**R**	**R**	S	S
	9	I	S	**R**	**R**	**R**	**R**	**R**	S	S	S	I	**R**	S	S	S	S	S	S	S	S	**R**	**R**	S	S
	10	**R**	**R**	**R**	**R**	**R**	**R**	**R**	S	S	S	**R**	S	**R**	**R**	**R**	**R**	**R**	**R**	**R**	**R**	**R**	**R**	**R**	**R**
	11	I	**R**	**R**	**R**	**R**	**R**	I	S	S	S	**R**	**R**	**R**	**R**	**R**	**R**	S	S	S	S	**R**	**R**	S	S
	13	S	S	I	S	**R**	**R**	**R**	S	S	S	I	**R**	I	S	S	S	S	S	S	S	**R**	**R**	**R**	S
	14	S	S	**R**	**R**	**R**	**R**	S	S	S	S	**R**	S	S	S	S	S	I	I	**R**	**R**	**R**	S	**R**	**R**
	20	**R**	S	**R**	**R**	**R**	S	I	S	**R**	S	**R**	S	**R**	**R**	**R**	**R**	**R**	**R**	S	S	**R**	S	I	**R**
Klebsiella pneumoniae (n=4)	6	S	S	**R**[a]	**R**[a]	**R**	S	S	S	S	S	**R**	**R**	S	S	S	S	S	S	**R**	**R**	**R**	**R**	I	**R**
	10	S	I	**R**[a]	**R**[a]	**R**	S	**R**	S	S	S	S	S	I	S	S	S	S	S	S	S	**R**	**R**	**R**	**R**
	12	I	I	**R**[a]	**R**[a]	**R**	**R**	**R**	**R**	S	S	S	S	I	I	S	S	S	S	**R**	**R**	**R**	**R**	**R**	**R**
	20	**R**	I	**R**[a]	**R**[a]	**R**	**R**	**R**	**R**	S	S	**R**	**R**	**R**	**R**	I	I	S	S	**R**	**R**	**R**	**R**	I	**R**
Enterobacter cloacae (n=1)	2	**R**	**R**	**R**[a]	nd	**R**	**R**	**R**	S	S	S	S	S	I	I	**R**	S	S	S	**R**	**R**	**R**	**R**	I	**R**

Amoxicillin-clavulanic acid (AMC); ampicillin (AMP); cefalexin (CL); cefovecin (CVN); imipenem (IPM); gentamicin (CN); enrofloxacin (ENR); marbofloxacin (MAR); chloramphenicol (C); sulfamethoxazole + trimethoprim(SXT); methicillin (MET); piperacillin (PIP); doxycycline (DO); tetracycline (TE); [a]Intrinsic resistance [32]; nd: not determined.
