# Supplementary material for: Antimicrobial Resistance (AMR) of Bacteria Isolated from Dogs with Canine Parvovirus (CPV) Infection: The Need for a Rational Use of Antibiotics in Companion Animal Health
Source: Antibiotics (Basel). 2022 Jan 23;11(2):142. doi: 10.3390/antibiotics11020142 (PMC8868125; doi:10.3390/antibiotics11020142)
Supplement: Supplementary file 1 [file antibiotics-11-00142-s001.zip › antibiotics-1500206-supplementary/Supplementary Material - Table S5.pdf]

Supplementary Material - Table S5. Comparison of the results obtained with the two methods for MDR Gram positive strains (n=2)

**Bacterial isolates**	**Dog id.**	Tetracyclines		Fluoroquinolones				Chloramphenicol		Lincosamides		Aminoglycosides		Sulfonamides		Macrolides	
		**DO**		**ENR**		**MAR**		**C**		**DA**		**CN**		**SXT**		**SP**	**E**
		KB	MIC	KB	MIC	KB	MIC	KB	MIC	KB	MIC	KB	MIC	KB	MIC	KB	MIC
Enterococcus faecium (n=1)	12	I	**R**	**R**	**R**	**R**	**R**	S	S	**R**[a]	nd	**R**[a]	nd	**R**[a]	nd	I	**R**
Staphylococcus xylosus (n=1)	4	S	**R**	**R**	**R**	S	**R**	S	S	**R**	**R**	S	S	S	S	**R**	I

Doxycycline (DO); enrofloxacin (ENR); marbofloxacin (MAR); chloramphenicol (C); clindamycin (DA); gentamicin (CN); sulfamethoxazole + trimethoprim (SXT); spiramycin (SP); erythromycin (E); [a]Intrinsic resistance [32]; nd: not determined.
