# Supplementary material for: Antimicrobial Resistance (AMR) of Bacteria Isolated from Dogs with Canine Parvovirus (CPV) Infection: The Need for a Rational Use of Antibiotics in Companion Animal Health
Source: Antibiotics (Basel). 2022 Jan 23;11(2):142. doi: 10.3390/antibiotics11020142 (PMC8868125; doi:10.3390/antibiotics11020142)
Supplement: Supplementary file 1 [file antibiotics-11-00142-s001.zip › antibiotics-1500206-supplementary/Supplementary Material - Table S6.pdf]

Supplementary Material - Table S6. Details on additional virologic tests

Virus	PCR	Kit	Primer reference	Reaction mix	Thermal conditions
CAdVs types 1 and 2	Single step	GoTaq G2 DNA Polymerase (Promega)	[74]	50-μl reaction mix: 10 μl of 5× GoTaq® Reaction Buffer, 1 μl of dNTP mix (10 mM), 0.25 μl of each primer CAV L and CAV R (0.1 μM), 0.25 μl of GoTaq® G2 DNA Polymerase, 33.25 μl of nuclease-free water and 5 μl of DNA extract	94°C for 2 min to activate TaqPol, followed by 32 cycles of 94°C for 30 s, 58°C for 1 min, 72°C for 1 min and a final extension of 72°C for 5 min
CDV	RT-PCR	OneStep RT-PCR Kit (Qiagen)	[73]	50-μl reaction mix: 10 μl of 5× RT-PCR Buffer, 2 μl of dNTP mix (10 mM), 1 μl of each primer DMV 1 and DMV 2 (0.4 μM), 0.22 μl of RNase Inhibitor (40 U/μl), 2 μl of QIAGEN OneStep RT-PCR Enzyme Mix, 31.28 μl of nuclease-free water and 2.5 μl of RNA extract	50°C for 30 min to synthesize the first cDNA, 95°C for 15 min to inactivate the reverse transcriptase, followed by 35 cycles of 94°C for 45 sec, 56°C for 45 sec, 72 °C for 1 min, and a final extension of 72 °C for 10 min
CCoV	RT-PCR	OneStep RT-PCR Kit (Qiagen)	[75]	50-μl reaction mix: 10 μl of 5× RT-PCR Buffer, 2 μl of dNTP mix (10 mM), 1 μl of each primer CCV1 and CCV2 (0.4 μM), 0.22 μl of RNase Inhibitor (40 U/μl), 2 μl of QIAGEN OneStep RT-PCR Enzyme Mix, 31.28 μl of nuclease-free water and 2.5 μl of RNA extract	50°C for 30 min to synthesize the first cDNA, 95°C for 15 min to inactivate the reverse transcriptase, followed by 40 cycles of 94°C for 1 min, 55°C for 1 min, 72 °C for 1 min, and a final extension of 72 °C for 10 min
CRoV	RNA denaturation			40 μl of RNA extract	97°C for 5 min

Reverse transcription	Taq DNA Polymerase PCR Buffer (Invitrogen); RANDOM Primers (Invitrogen); MMLV Reverse Transciptase (Invitrogen)		70-µl reaction mix: 7 µl of 10× PCR Buffer minus Mg, 7 µl of MgCl2 (50 mM), 2 µl of dNTP mix (10 mM), 1 µl of RANDOM Primers, 2 µl of M-MLV, 11 µl of nuclease-free water and 40 µl of denatured RNA	37°C for 60 min to synthesize the first cDNA, 95°C for 5 min to inactivate the reverse transcriptase
Real-Time PCR	TaqMan Universal PCR Master Mix (Applied Biosystem)	[76]	25-µl reaction mix: 12.5 µl of TaqMan Universal PCR Master Mix 2×, 1 µl of each primer NVP3-F Deg – Forward and NVP3-R1 - Reverse (5 µM), 1 µl of NVP3-Probe (FAM-MGB) (3.75 µM), 7.5 µl of nuclease-free water and 2 µl of cDNA	50°C for 2 min to activate TaqPol, 95°C for 10 min for preheating, followed by 40 cycles of 94°C for 20 sec and 60°C for 1 min
